# Supplementary material for: Laurequinone, a Lead Compound against Leishmania
Source: Mar Drugs. 2023 May 30;21(6):333. doi: 10.3390/md21060333 (PMC10304164; doi:10.3390/md21060333)
Supplement: Supplementary file 1 [file marinedrugs-21-00333-s001.zip › marinedrugs-2396793-supplementary.pdf]

Supporting Information

Laurequinone, a lead compound against *Leishmania*

Sara García-Davis[1,2,#], Atteneri López-Arencibia[3,4,5,6,#,*], Carlos J. Bethencourt-Estrella[3,4,6], Desirée San Nicolás-Hernández[3,4,6], Ezequiel Viveros-Valdez[7], Ana R. Díaz-Marrero[1,8], José J. Fernández[1,2], Jacob Lorenzo-Morales[3,4,5,6], José E. Piñero[3,4,5,6]

[1] Instituto Universitario de Bio-Orgánica Antonio González (IUBO AG), Universidad de La Laguna (ULL), Avenida Astrofísico Francisco Sánchez 2, 38206 La Laguna, Tenerife, Spain
[2] Departamento de Química Orgánica, Universidad de La Laguna, Avenida Astrofísico Francisco Sánchez 2, 38206 La Laguna, Tenerife, Spain
[3] Instituto Universitario de Enfermedades Tropicales y Salud Pública de Canarias, Universidad de La Laguna, Avenida Astrofísico Francisco Sánchez S/N, 38206 La Laguna, Tenerife, Islas Canarias, Spain
[4] Consorcio Centro de Investigación Biomédica en Red M.P. de Enfermedades Infecciosas (CIBERINFEC), Instituto de Salud Carlos III, 28006 Madrid, Spain
[5] Departamento de Obstetricia y Ginecología, Pediatría, Medicina Preventiva y Salud Pública, Toxicología, Medicina Legal y Forense y Parasitología, Universidad de La Laguna, Tenerife, Spain
[6] Red de Investigación Cooperativa en Enfermedades Tropicales (RICET), Spain
[7] Facultad de Ciencias Biológicas, Universidad Autónoma de Nuevo León, Avenida Pedro de Alba S/N, San Nicolás de los Garza 66450, Nuevo León, Mexico
[8] Instituto de Productos Naturales y Agrobiología (IPNA), Consejo Superior de Investigaciones Científicas (CSIC), Avenida Astrofísico Francisco Sánchez 3, 38206 La Laguna, Tenerife, Spain

Physical data of laurequinone (**1**)

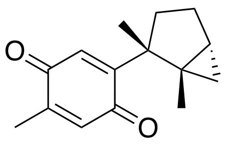

Laurequinone (**1**)

Yellow oil; $[\alpha]^{25}_{D}$ –29 (*c* 0.12, CH_2Cl_2)

HRESIMS *m/z* 229.1231 $[M\text{-}H]^-$ (calc. $C_{15}H_{17}O_2$, 229.1229)

^{1}H NMR (600 MHz, $CDCl_3$) δ 0.45 (2H, m, H-12), 1.07 (2H, m, H-3), 1.18 (1H, m, H-5), 1.18 (3H, s, H-15), 1.32 (3H, s, H-14), 1.66 (1H, d, J= 12.4, 8.1 *Hz*, H-4), 1.94 (1H, ddt, J= 15.7, 7.8, 4.1 *Hz*, H-4), 2.02 (3H, d, J= 1.6 *Hz*, H-13), 2.05 (1H, dd, J= 13.4, 8.2 *Hz*, H-5), 6.51 (1H, t, J= 1.6 *Hz*, H-11), 6.87 (1H, s, H-8)

^{13}C NMR (150 MHz, $CDCl_3$) δ 15.0 (C-12), 15.6 (C-13), 17.8 (C-15), 22.9 (C-14), 23.5 (C-3), 25.2 (C-4), 28.7 (C-2), 35.3 (C-5), 48.2 (C-1), 131.7 (C-8), 135.0 (C-11), 144.2 (C-9), 154.2 (C-6), 188.6 (C-7), 189.2 (C-10).

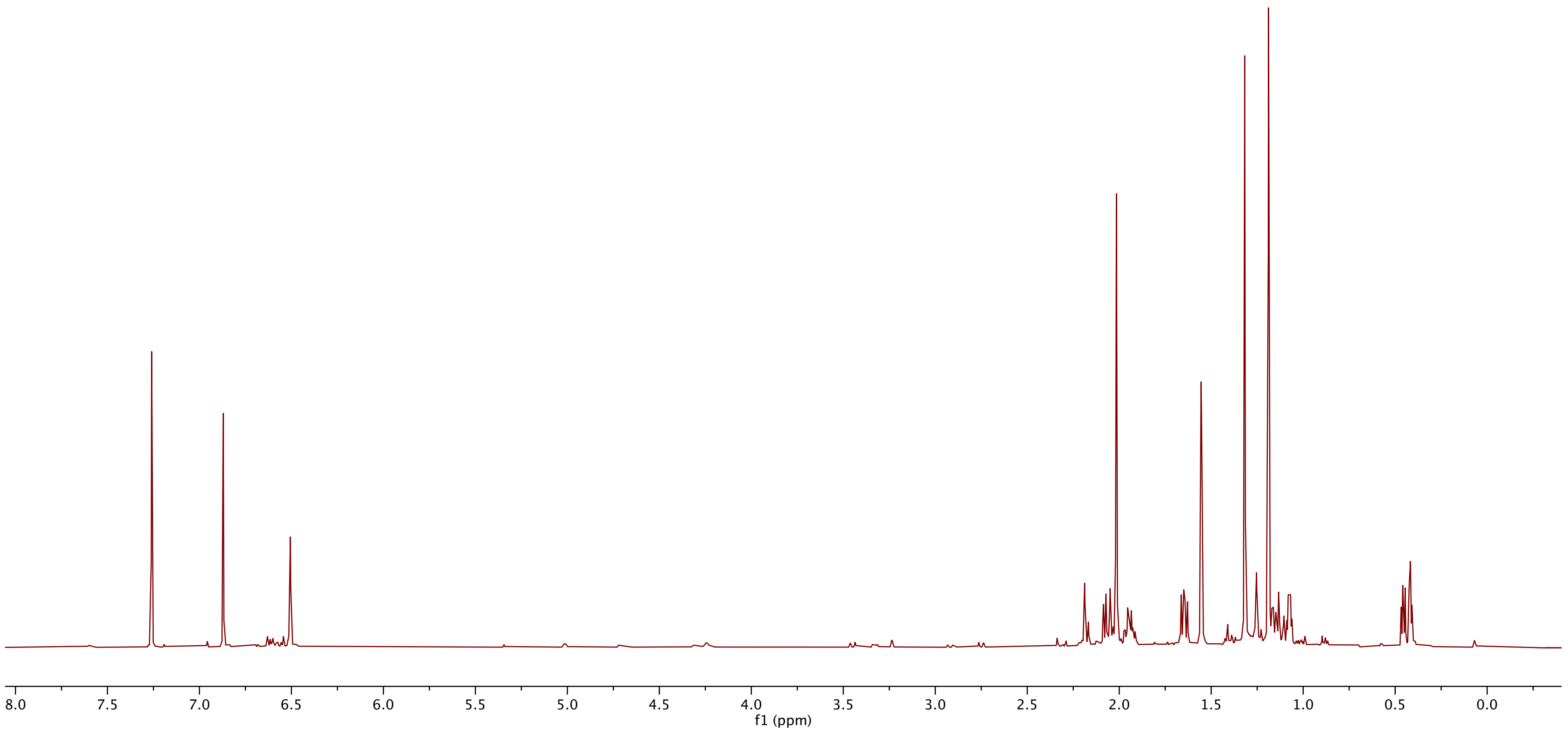

Figure S1: ^{1}H-NMR spectrum of laurequinone (**1**) at 600 MHz in $CDCl_3$

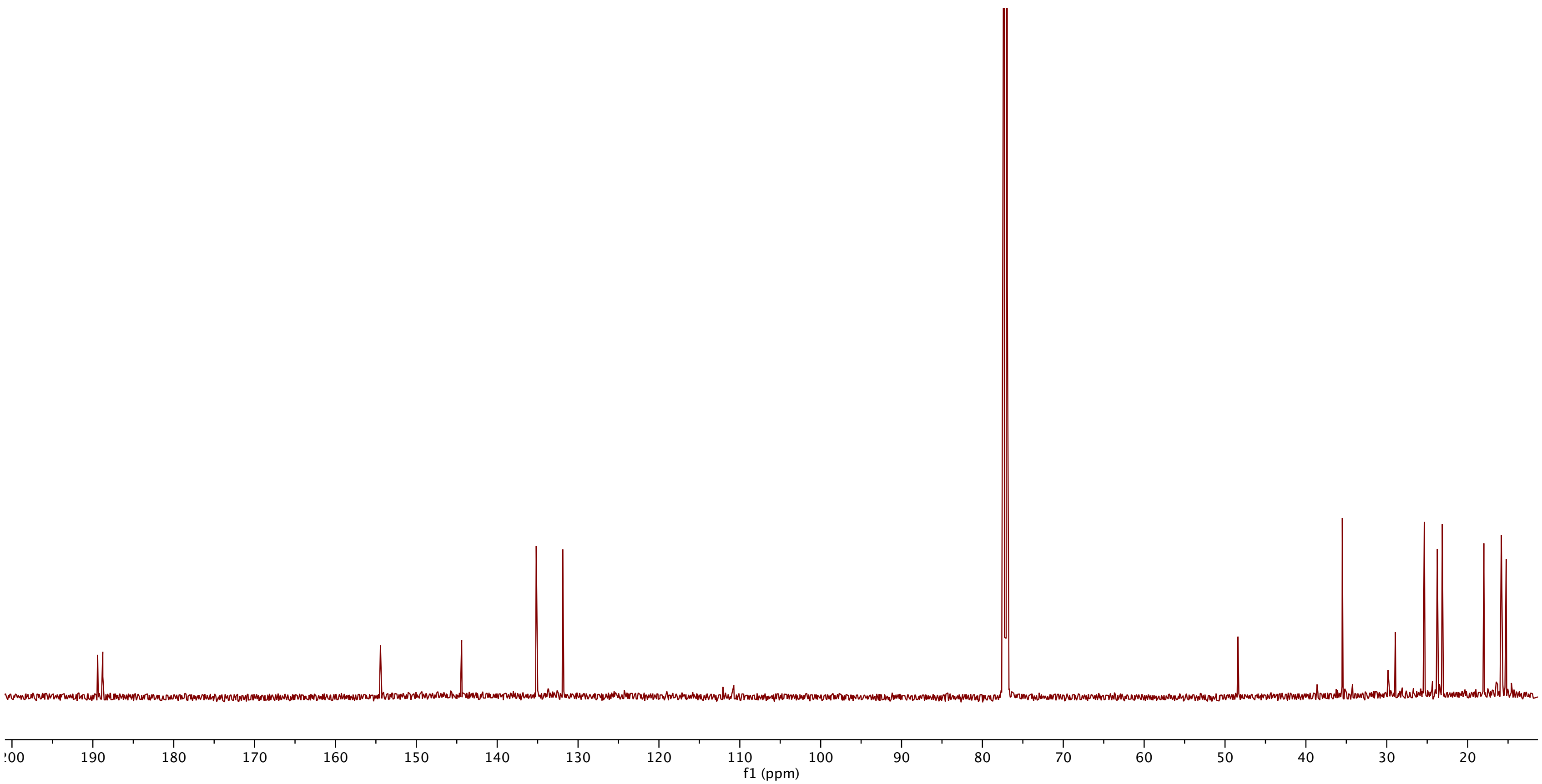

Figure S2: ^{13}C-NMR spectrum of laurequinone (**1**) at 150 MHz in $CDCl_3$

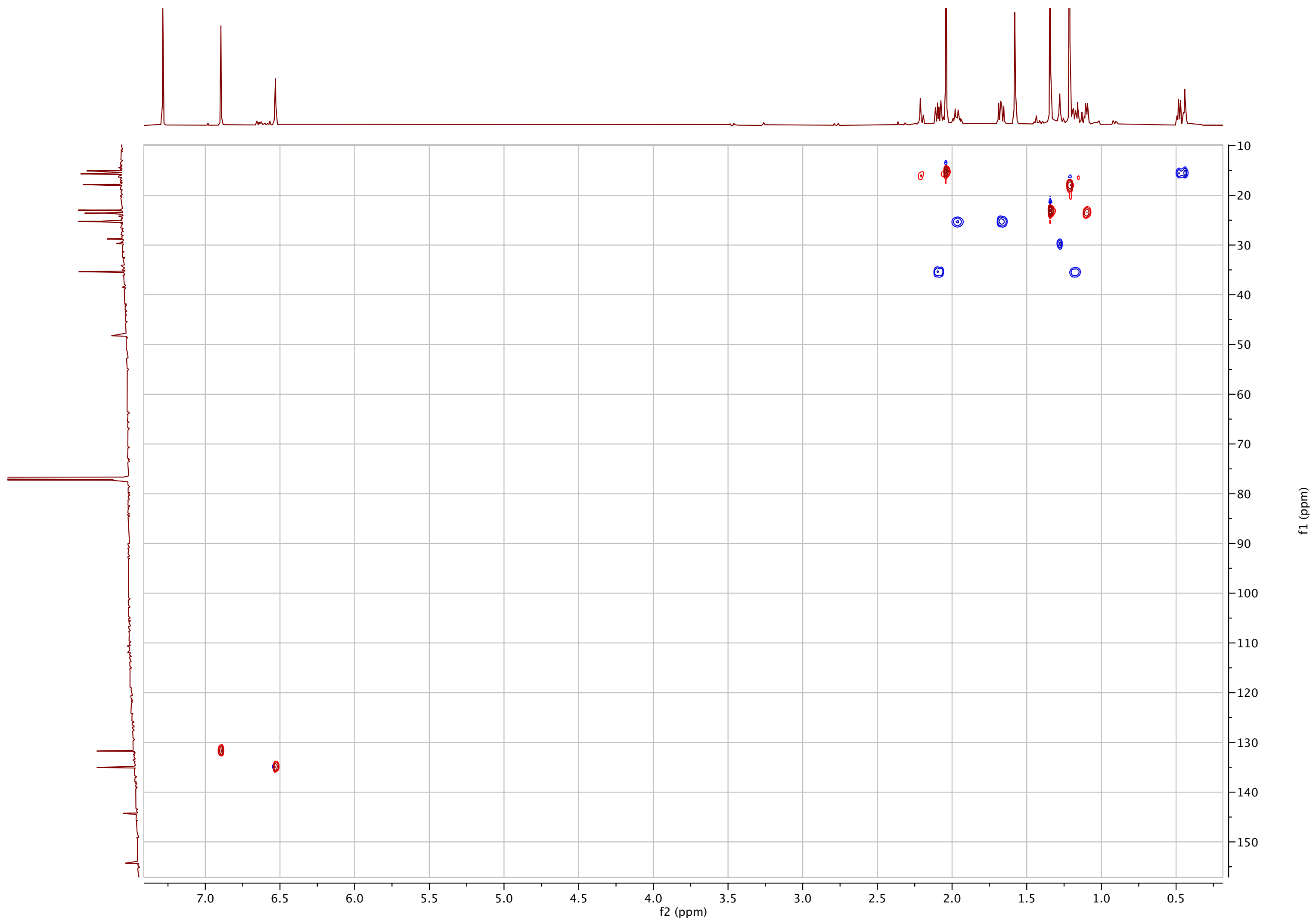

Figure S3: HSQC experiment of laurequinone (**1**) at 600 MHz in $CDCl_3$

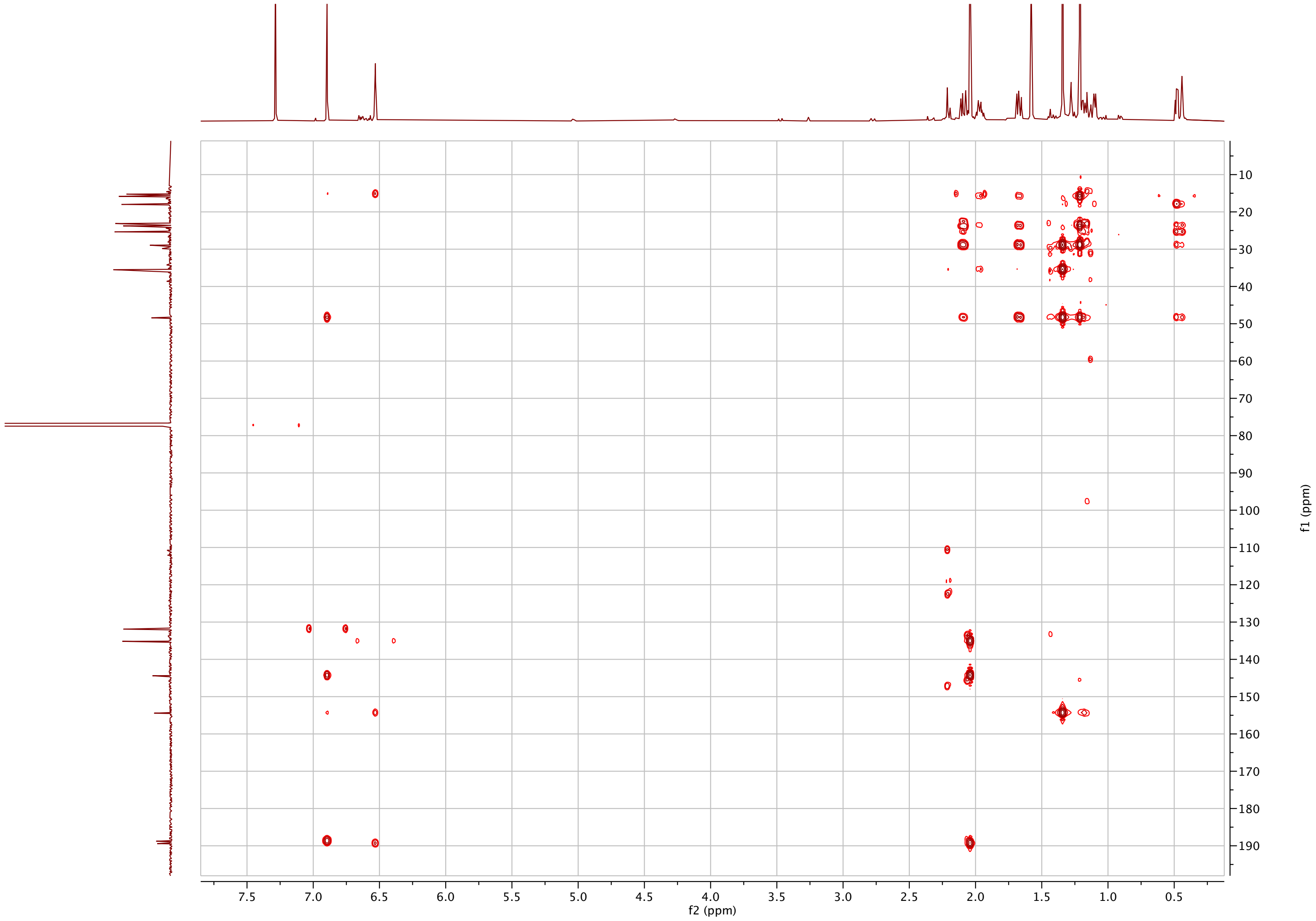

Figure S4: HMBC experiment of laurequinone (**1**) at 600 MHz in $CDCl_3$
